# Colchicine reduces atherosclerotic plaque vulnerability in rabbits

**DOI:** 10.1016/j.athplu.2021.08.008

**Published:** 2021-09-02

**Authors:** François Roubille, Nolwenn Merlet, David Busseuil, Marine Ferron, Yanfen Shi, Teodora Mihalache-Avram, Mélanie Mecteau, Geneviève Brand, Daniel Rivas, Mariève Cossette, Marie-Claude Guertin, Eric Rhéaume, Jean-Claude Tardif

**Affiliations:** a Montreal Heart Institute, Montreal, Quebec, Canada; b Department of Medicine, Université de Montréal, Montreal, Quebec, Canada; c Montreal Health Innovations Coordinating Centre (MHICC), Montreal, Canada

**Keywords:** Colchicine, Inflammation, Atherosclerosis, Plaque vulnerability, Vascular remodelling, Monocyte activation

## Abstract

**Background and aims:**

The anti-inflammatory agent colchicine is gaining interest as a treatment for coronary artery disease. However, the effects of colchicine in atherosclerotic animal models are mostly unknown. This study aimed to evaluate colchicine in a rabbit model of atherosclerosis.

**Methods:**

Twenty-two rabbits were fed a 0.5% cholesterol-enriched diet for 10 weeks and then randomized to receive either oral saline (n=11) or colchicine (350 μg/kg/day; n=11) for 6 weeks, with 0.2% cholesterol-diet during the treatment period. We performed intravascular ultrasound imaging (at start and end of treatment) and histology analyses of the descending thoracic aorta. Leucocyte activation was assessed *in vitro* on blood samples obtained during treatment.

**Results:**

Colchicine prevented positive aortic vascular remodelling (*p*=0.029 *vs* placebo). This effect was even more marked at high plasma cholesterol level (third quartile of plasma cholesterol, *p*=0.020). At high cholesterol level, both atherosclerotic plaque and media areas on histomorphology were reduced by colchicine compared to placebo (*p*=0.031 and *p*=0.039, respectively). Plaque fibrosis and macrophage area were reduced by colchicine (Masson's trichrome stain: *p*=0.038; RAM-11: *p*=0.026). The plaque vulnerability index, assessed by histology, was reduced by colchicine (*p*=0.040). Elastin/type I collagen ratio in media was significantly higher with colchicine compared to placebo (*p*=0.013). At a high level of plasma cholesterol, *in vitro* LPS challenge revealed a decrease in monocyte activation following treatment with colchicine (*p*<0.001) and no change in the placebo group (*p*=0.353).

**Conclusions:**

Colchicine decreases plaque vulnerability with reductions in plaque inflammation, medial fibrosis, outward vascular remodelling and *ex vivo* monocyte activation.

## Highlights

• Colchicine decreases plaque vulnerability and outward vascular remodelling.
• Colchicine decreases plaque inflammation and medial fibrosis.
• Colchicine reduces circulating monocyte activation by LPS.

## Graphical abstract

**Figure:**
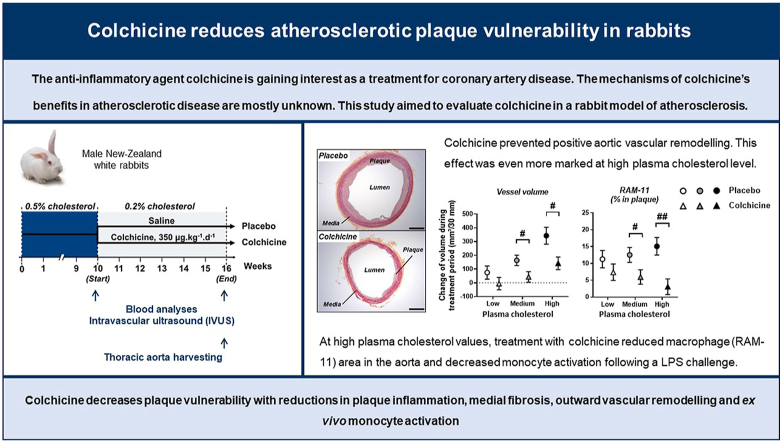

## 1 Introduction

Despite intensive risk factor modification and optimal medical therapy including with statins, anti-platelet and anti-hypertensive medications [1,2], atherosclerosis remains one of the main processes underlying cardiovascular diseases, which in turn are the leading cause of death worldwide [3]. Atherosclerosis is a complex inflammatory disease that involves several cell types, chemokines, cytokines and adhesion molecules [4]. Over the last two decades, both basic studies and clinical trials have provided evidence for the potential benefits of selectively reducing inflammation in atherosclerosis [5]. More recently, in the CANTOS (Canakinumab Anti-Inflammatory Thrombosis Outcomes Study) trial the anti-interleukin (IL)-1β antibody canakinumab was shown to reduce the risk of cardiovascular events in patients with coronary disease receiving standard of care [6]. However, that agent requires regular injections, is very costly and blocks only one of many inflammatory pathways. The search for less expensive agents, potentially with a wider anti-inflammatory spectrum, is ongoing. In contrast to methotrexate in CIRT (Cardiovascular Inflammation Reduction Trial) that did not provide cardiovascular benefits in patients with atherosclerotic vascular disease [7], the interest for colchicine has been growing [8,9].

Colchicine is a widely available, inexpensive, orally administered, potent anti-inflammatory medication that is approved for the management of patients with gout, familial Mediterranean fever and pericarditis [10]. The active compound, initially extracted from the plant autumn crocus (*Colchicum autumnale*), has been used for centuries and is one of the oldest drugs still currently available. The main mechanism of action by which colchicine exerts its effects is through the inhibition of tubulin polymerization and subsequent microtubule generation and potentially also through effects on cellular adhesion molecules and inflammatory chemokines [10]. Through its action on tubulin, colchicine can interfere with many functions of white blood cells including migration and degranulation. Colchicine may have other direct anti-inflammatory effects by inhibiting key inflammatory signaling networks such as the inflammasome and pro-inflammatory cytokines. It has been reported to inhibit membrane expression of adhesion molecules on T cells and endothelial cells. Colchicine also exerts a direct anti-inflammatory effect by inhibiting the synthesis of tumor necrosis factor alpha and IL-6, the migration of monocytes, and the secretion of matrix metalloproteinase-9. Through the disruption of the cytoskeleton, colchicine is believed to suppress secretion of cytokines and chemokines.

In the LoDoCo trial, 532 patients with coronary disease were randomly assigned to receive treatment with colchicine or no colchicine in addition to usual care. Following a mean follow-up of 36 months, colchicine-treated patients experienced significantly fewer cardiovascular events as compared with those not treated with colchicine [11]. However, that study was relatively small and not placebo-controlled. Since then, several large-scale, rigorous trials have been conducted, including COLCOT (Colchicine Cardiovascular Outcomes Trial) and LoDoCo2 (Low-Dose Colchicine-2) where treatment with 0.5 mg colchicine daily in comparison with placebo provided large relative risk reductions of ischemic cardiovascular events in adults who had experienced a myocardial infarction or had chronic coronary disease [12,13]. Nevertheless, the underlying mechanism of action remains obscure and little is known about the effects of colchicine on atherosclerosis. Therefore, the aim of the present study was to evaluate the effects of colchicine on plaque characteristics such as inflammation, fibrosis and vulnerability as well as accompanying vascular remodelling in a rabbit model of atherosclerosis.

## 2 Material and methods

### 2.1 Animal studies

Animal care and procedures complied with the Canadian Council on Animal Care guidelines and were approved by the institutional ethics committee for animal research. Twenty-three (23) male New-Zealand White rabbits (2.35 ± 0.07 kg) were purchased from Charles River (St-Constant, Canada). Rabbits were fed a 0.5% cholesterol-enriched diet (Teklad Global Rabbit Diet 2030, *Harlan Laboratories*, Madison, WI) for 10 weeks and then randomized to treatment with colchicine 350 μg/kg/day (based on previous rodent studies [14,15], n=11) or saline (placebo group, n=11). Treatment was administered orally (mixed in yogurt) once daily, 5 days per week, for 6 weeks. During the randomized treatment period, a 0.2% cholesterol-enriched diet (Teklad Global Rabbit Diet 2030, *Harlan Laboratories*, Madison, WI) was used to mimic cholesterol-lowering therapy in both groups. At start and end of treatment (weeks 10 and 16, respectively), rabbits were imaged by intravascular ultrasound (IVUS, see details in Supplementary methods). Blood samples were obtained through the ear marginal vein at weeks 0 (baseline), 10, 12, 14 and 16 under acepromazine tranquilization (1 mg/kg, i.m.). White blood cell counts were determined by a Coulter Counter (*Beckman Coulter*). After the final evaluation (IVUS imaging at week 16), rabbits were sacrificed by exsanguination under anaesthesia. The aorta was harvested and embedded in tissue freezing medium (*Thermo Scientific*) and frozen at −80°C for histological analyses.

### 2.2 Histology and histomorphometry

Thoracic aorta cross-sections (8 μm) were stained with Masson's trichrome to assess fibrosis, Movat's pentachrome for elastin, Oil-Red-O for lipid droplets, von Kossa for plaque calcification and haematoxylin-phloxin-safran (HPS) to determine lumen area, external and internal elastic lamina (EEL and IEL, respectively) areas. Media area was calculated as EEL area *minus* IEL area, and thoracic aorta plaque area was calculated as IEL area *minus* lumen area.

All measurements were performed by two experienced investigators blinded to randomized treatment assignment.

### 2.3 Immunohistochemistry

We assessed the levels of macrophages (RAM-11^+^ cells), T-cells (CD3^+^ cells), neutrophils (cells positive for monoclonal RPN3/57 antibody), vascular smooth muscle cells (vSMCs; SM-α-actin^+^ cells, SM myosin heavy chain I [SM1] and II [SM2]), as well as various markers of inflammation (interleukin 6 [IL-6], interleukin 1 beta [IL-1β], Nucleotid binding oligomerization domain-like receptor family pyrin domain containing 3 [NLRP3], vascular cell adhesion molecule 1 [VCAM-1 or CD106], intercellular adhesion molecule 1 [ICAM-1 or CD54], proprotein convertase subtilisin/kexin type 9 [PCSK9]) and fibrosis (types I and III collagen, matrix metalloproteinase 9 [MMP9], transforming-growth factor beta1 [TGF-β_1_], fibronectin, and phospho-SMAD2/3) on thoracic aorta cross-sections.

The atherosclerotic plaque vulnerability index was calculated as (% RAM-11 staining + % ORO staining) / (% α-SM-actin staining + % type I and % type III collagen staining) [16,17].

All measurements were performed by two experienced investigators blinded to randomized treatment assignment.

### 2.4 Statistical analyses

Statistical analyses were performed using SAS statistical software version 9.3 or higher (SAS Institute Inc., Cary, NC, USA) and conducted at the 0.05 significance level.

See additional details in the Supplementary document.

## 3 Results

### 3.1 Colchicine 350 μg/kg/day is well tolerated by atherosclerotic rabbits

No adverse reaction to colchicine occurred in rabbits during the 6-week treatment period: body weights were similar between placebo and colchicine groups (*p*=0.335; Supplementary Fig. S2) and neither diarrhea nor alopecia was observed. There were also no difference between groups for change in lipid profiles (Supplementary Table S2), white blood cell counts (lymphocytes: *p*=0.425; monocytes: *p*=0.630; granulocytes: *p*=0.622), liver enzymes (ALT: *p*=0.545; AST: *p*=0.538; GGT: *p*=0.320) and creatinine (*p*=0.572).

### 3.2 Colchicine reduces aortic positive (outward) vascular remodelling

**Fig. 1:**
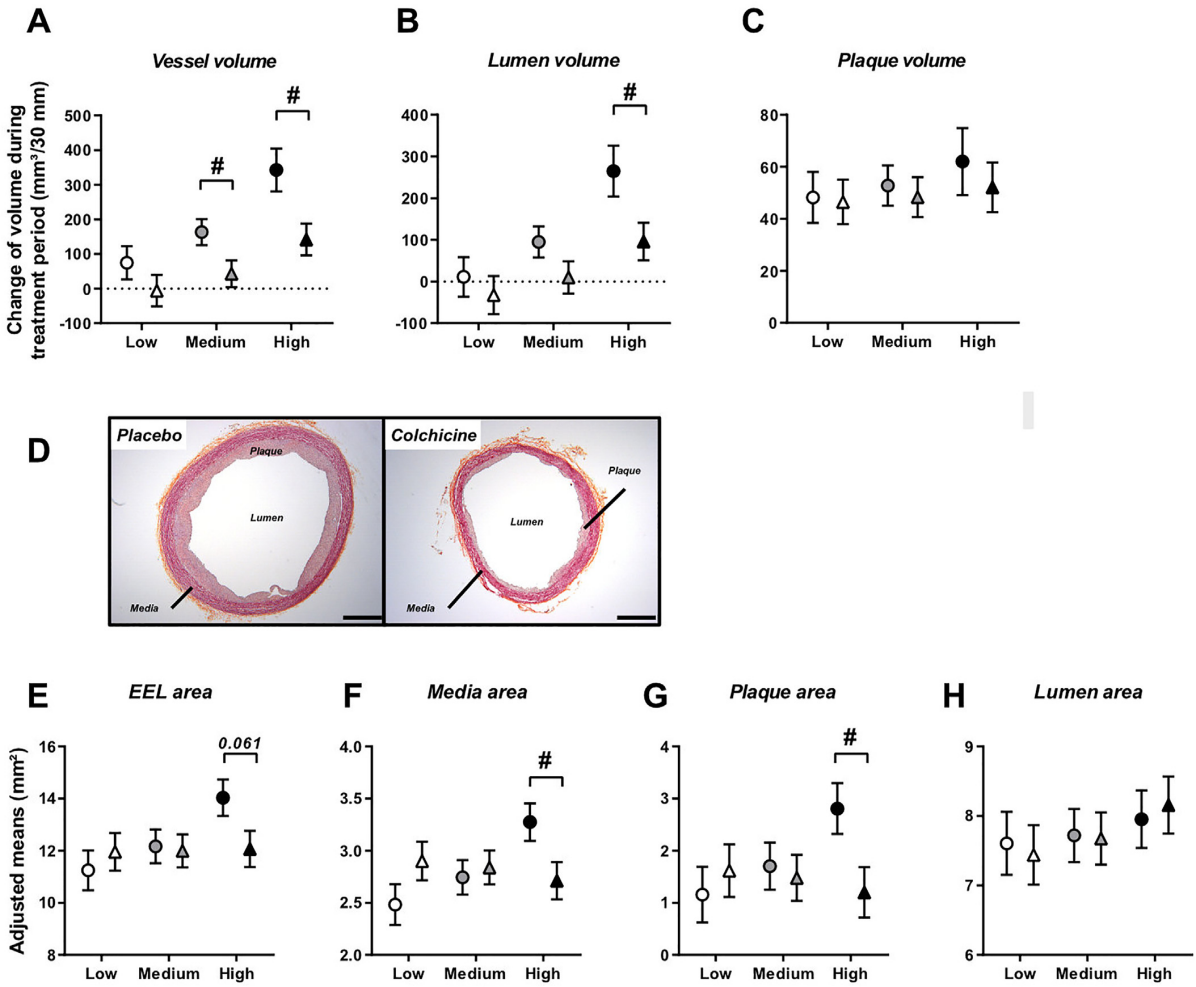
Colchicine reduces aortic outward remodelling as assessed by IVUS and histomorphometric analyses Plots in (A, B, C) represent adjusted mean changes ± SE in total vessel volume, lumen volume and plaque volume of thoracic aorta, respectively, from weeks 10–16 for placebo (circles, n=10) and colchicine-treated (triangles, n=10) groups at specific values of plasma cholesterol at start of treatment (low, medium and high). Pictures in (D) represent descending thoracic aorta cross sections (8 μm) of placebo and colchicine-treated rabbits, stained with haematoxylin-phloxin-safran (HPS) to assess media and plaque areas (magnification x2; scale bars indicate 1 mm). Plots in (E–H) represent adjusted means ± SE of external elastic lamina (EEL), media, plaque and lumen areas in descending thoracic aorta assessed on HPS-stained sections, for placebo (circles, n=11) and colchicine-treated (triangles, n=11) groups at specific values of plasma cholesterol at start of treatment (low, medium and high). ^**#**^*P*<0.05, for colchicine *vs.* placebo.

Aortic remodelling was assessed in the descending thoracic aorta by both IVUS and histomorphometry analyses (Fig. 1). On IVUS, adjusted mean change in total vessel volume from weeks 10–16 showed a significant increase in the placebo group (173 ± 38 mm³, corresponding to a 26.7% increase; *p*<0.001) but not in the colchicine group (41 ± 38 mm³, corresponding to a non-significant 6.3% change; *p*=0.299), resulting in a significant difference between groups (*p*=0.029). When exploring comparisons between groups at specific values of plasma cholesterol at start of treatment, this colchicine effect was mainly observed at medium and high levels (*p*=0.254 at low cholesterol level; *p*=0.044 at medium cholesterol level; *p*=0.020 at high cholesterol level; Fig. 1A). A similar pattern was observed for lumen volume, where adjusted mean change from weeks 10–16 showed an increase with placebo (104 ± 38 mm³, *p*=0.015) but not with colchicine (5 ± 38 mm³, *p*=0.896; *p*=0.093 between groups). This difference was significant at high cholesterol level at start of treatment (*p*=0.041; Fig. 1B). Plaque volume increased both in the placebo (55 ± 7 mm³, *p*<0.001) and colchicine (50 ± 7 mm³, *p*<0.001) groups (*p*=0.659 between groups), irrespective of plasma cholesterol level at start of treatment (*p*=0.895, 0.689 and 0.551 at low, medium and high cholesterol levels, respectively; Fig. 1C).

The *in vivo* IVUS imaging results demonstrating differences in vascular remodelling between groups were supported by histomorphological analyses, even if the latter were performed on thoracic descending aortas that were not fixed under pressure. Histomorphometry showed a numerically smaller EEL area in the colchicine group (12.1 ± 0.7 mm^2^) compared to the placebo group (14.0 ± 0.7 mm^2^, *p*=0.061 between groups) at high cholesterol level at start of treatment, but not at low and medium levels (*p*=0.507 and *p*=0.850, respectively; Fig. 1D and E). Atherosclerotic media area and plaque area were smaller with colchicine compared to placebo (2.71 ± 0.18 mm^2^ *vs.* 3.27 ± 0.18 mm^2^, *p*=0.039; and 1.20 ± 0.48 mm^2^ *vs.* 2.81 ± 0.49 mm^2^, *p*=0.031, respectively) at high cholesterol level at start of treatment, whereas no difference was observed between groups at low (*p*=0.138 and *p*=0.540) or medium levels (*p*=0.688 and *p*=0.727; Fig. 1D, F and G). Lumen area was similar between both treatment groups (*p*=0.792, 0.935 and 0.731 at low, medium and high cholesterol levels, respectively; Fig. 1D and H).

### 3.3 Colchicine reduces plaque macrophage infiltration and medial fibrosis

**Fig. 2:**
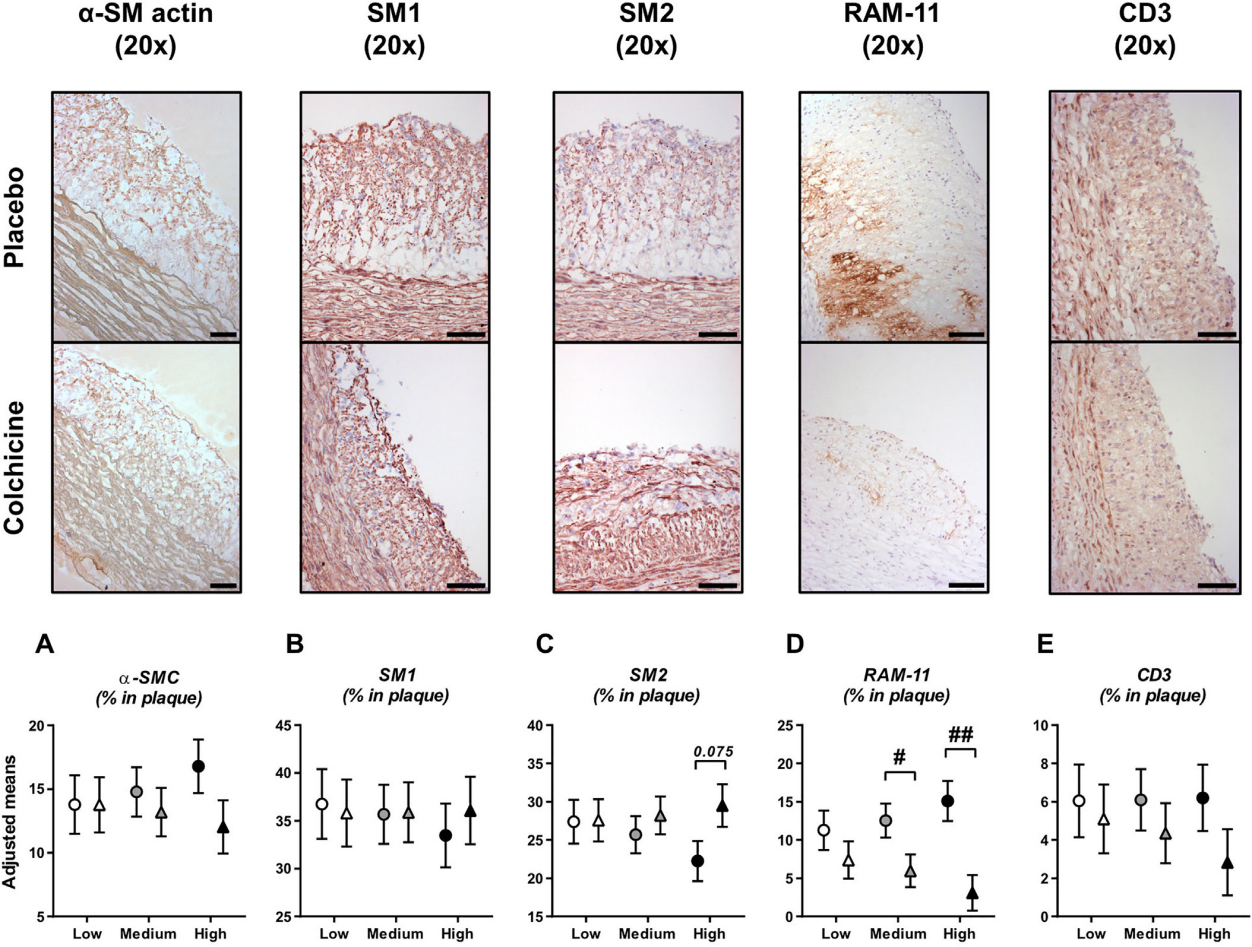
Colchicine reduces plaque macrophages Plots in (A–E) represent cellular composition of atherosclerotic plaques assessed by presence of vascular smooth muscle cells (vSMCs, α-SM-actin positive staining area), macrophages (RAM-11^+^ cells) and T-cells (CD3^+^ cells). Phenotype of vSMCs was further characterized by SM1 and SM2 markers. Results are expressed as adjusted means ± SE of each staining for placebo (circles, n=11) and colchicine-treated (triangles, n=11) groups at specific values of plasma cholesterol at start of treatment (low, medium and high). Pictures are representative stainings at high plasma cholesterol value at start of treatment. Scale bars represent 100 μm. ^**#**^*P*<0.05, ^**##**^*P*<0.01, for colchicine *vs.* placebo.

To understand the mechanisms underlying colchicine's effect on vascular remodelling, cellular composition and extracellular matrix elements of atherosclerotic plaques were assessed by several immunohistochemistry and histological stainings performed on thoracic aorta cross-sections (Fig. 2). The area occupied by vascular smooth muscle cells was similar in both experimental groups (*p*=0.994, 0.566 and 0.126 at low, medium and high cholesterol levels, respectively; Fig. 2A). SM1 marker expression was similar between groups whereas a trend for higher SM2 expression in the colchicine group compared to placebo was observed at high cholesterol level at start of treatment (29.5 ± 2.8% *vs.* 22.3 ± 2.6%, *p=*0.075), but not at low and medium cholesterol levels (*p*=0.965 and 0.477, respectively; Fig. 2B, C). The abundance of plaque macrophages (RAM-11^+^ cells) was reduced by colchicine compared to placebo (5.1 ± 2.3 *vs.* 13.1 ± 2.4%, *p*=0.026). This decrease in plaque macrophages was mainly observed at medium and high cholesterol levels at start of treatment (6.0 ± 2.1% *vs.* 12.5 ± 2.2%, *p*=0.047 at medium cholesterol level; and 3.1 ± 2.3% *vs.* 15.1 ± 2.6%, *p*=0.003 at high cholesterol level), but not at lower cholesterol level (*p*=0.287; Fig. 2D). T-cells (CD3^+^) in plaques were similar between groups (*p*=0.722, 0.447 and 0.186 at low, medium and high cholesterol levels, respectively; Fig. 2E). We could not detect neutrophils in atherosclerotic plaques.

Lipid infiltration (ORO staining) and calcification of the atherosclerotic plaque (Von Kossa staining) were not different between groups (respectively: *p*=0.257, *p*=0.902; Supplementary Figs. S3A and S3B). Plaque fibrosis assessed by Masson's trichrome staining was reduced in the colchicine group compared to placebo (12.0 ± 2.0% *vs.* 18.4 ± 2.0%, *p*=0.038). This decrease in fibrosis with colchicine was mainly observed at high (11.0 ± 2.2% *vs.* 18.8 ± 2.3%, *p*=0.027) and medium (12.6 ± 2.1% *vs.* 18.3 ± 2.1%, *p*=0.075) cholesterol level at start of treatment, but not at low level (*p*=0.198; Supplementary Fig. S3C). Several fibrosis markers (types I and III collagen, MMP9, TGF-β_1_, fibronectin, P-SMAD 2/3) were also assessed in the plaque but their expression was not significantly different between groups (Supplementary Figs. S3D–I).

**Fig. 3:**
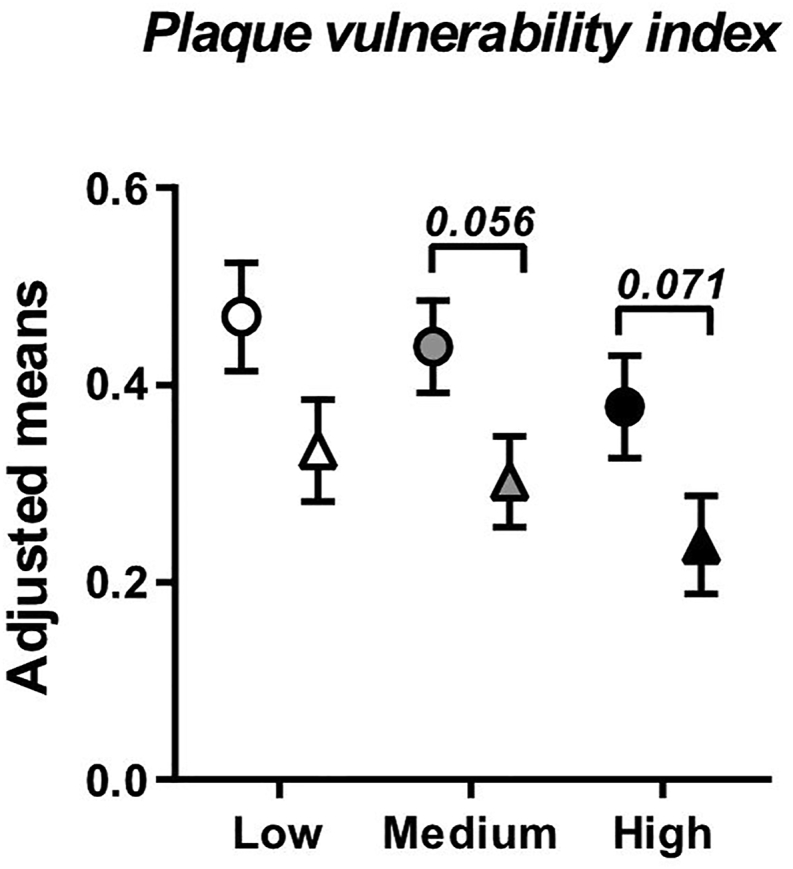
Colchicine reduces plaque vulnerability index Plaque vulnerability index was calculated as (% RAM-11 staining + % ORO staining) / (% α-SM-actin staining + % type I and % type III collagen staining). Results are expressed as adjusted means ± SE for placebo (circles, n=11) and colchicine-treated (triangles, n=11) groups at specific values of plasma cholesterol at start of treatment (low, medium and high).

The atherosclerotic plaque vulnerability index includes the factors associated with increasing (lipids, inflammatory markers) and decreasing (SMCs, fibrosis markers) plaque instability and was calculated as (% RAM-11 staining + % ORO staining) / (% α-SM-actin staining + % type I and % type III collagen staining). Adjusted mean of plaque vulnerability index was significantly lower with colchicine compared to placebo (0.281 ± 0.043 *vs.* 0.419 ± 0.043, *p*=0.040). This effect of colchicine was mainly observed at medium and high plasma cholesterol levels (*p*=0.095, 0.056 and 0.071 at low, medium and high cholesterol levels; Fig. 3).

**Fig. 4:**
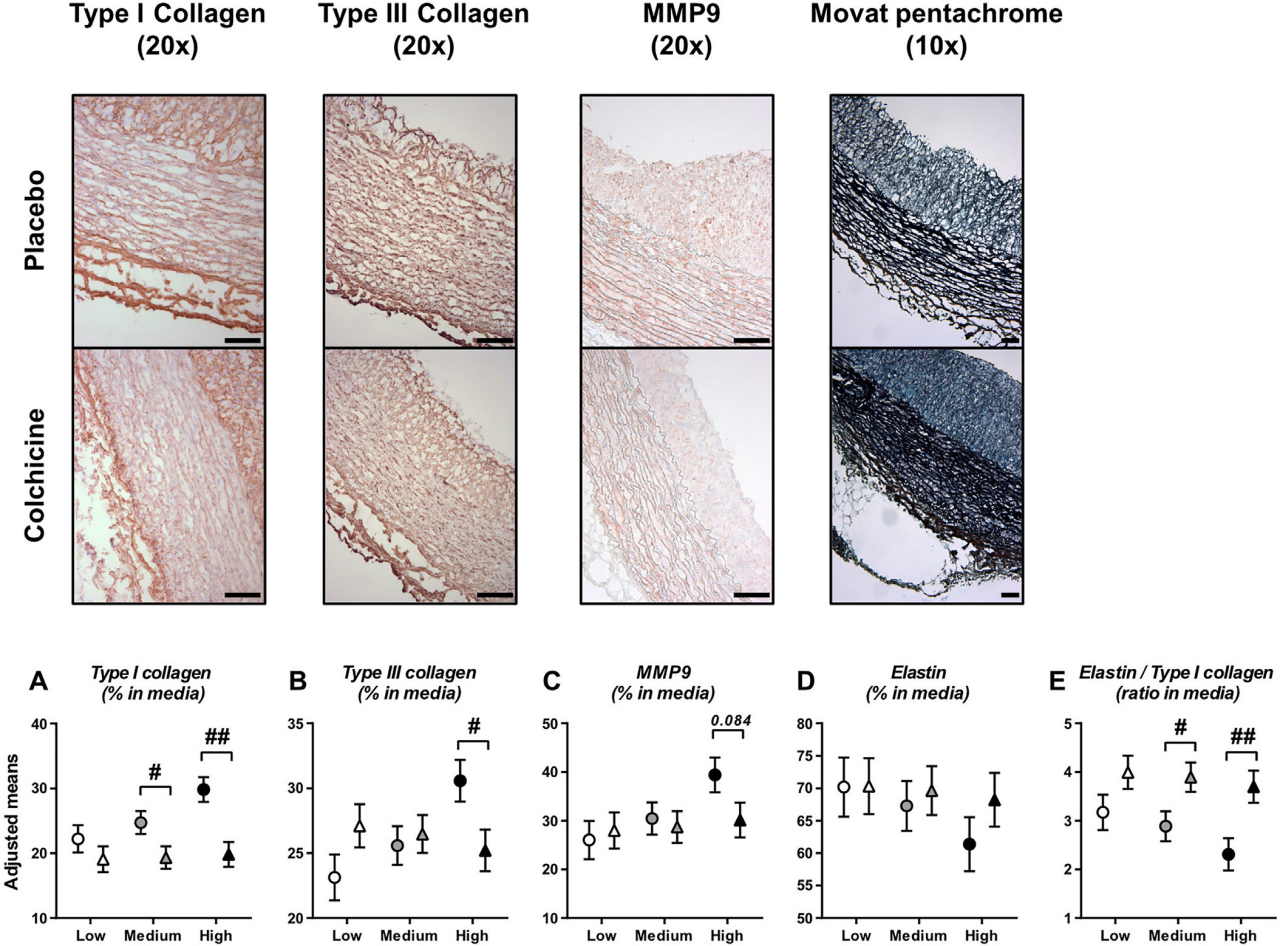
Colchicine reduces medial fibrosis Plots in (A–D) represent types I and III collagen, matrix metalloproteinase 9 (MMP9) and elastin expression assessed in aortic media. Results are expressed as adjusted means ± SE of each staining for placebo (circles, n=11) and colchicine-treated (triangles, n=11) groups at specific values of plasma cholesterol at start of treatment (low, medium and high). The elastin/type I collagen ratio was calculated as an index of aortic elasticity (E). Pictures are representative stainings at high plasma cholesterol value at start of treatment. Scale bars represent 100 μm. ^**#**^*p*<0.05, ^**##**^*p* < 0.01, for colchicine *vs.* placebo.

Key components of vessel media (collagen and elastin) were investigated. Significant differences between groups were observed for both types I and III collagen in media at high plasma cholesterol level (19.8 ± 1.9% for colchicine *vs.* 29.8 ± 1.9% for placebo, *p*=0.002; and 25.2 ± 1.6% *vs.* 30.6 ± 1.6% *p*=0.029; Fig. 4A, B). MMP9 expression tended to be lower in colchicine-treated rabbits compared to placebo at high plasma cholesterol level (30.2 ± 3.6% *vs.* 39.4 ± 3.6%, *p*=0.084; Fig. 4C). Elastin expression in media was similar between both treatment groups (*p*=0.462), but the elastin/type I collagen ratio was significantly higher with colchicine compared to placebo (3.82 ± 0.29 *vs.* 2.68 ± 0.29, *p*=0.013). This difference was mainly observed at medium and high plasma cholesterol levels at start of treatment (*p*=0.117, 0.030 and 0.008 at low, medium and high levels, respectively; Fig. 4D, E).

### 3.4 Colchicine does not reduce inflammatory marker levels in plaques

As colchicine reduced macrophage infiltration, we analyzed inflammatory marker expression (IL-6, IL-1β, NLRP3, VCAM-1) in plaques, but none of them were different between treatment groups (Supplementary Figs. S4A–D). ICAM-1 (also known as CD54) was higher with colchicine compared to placebo (3.91 ± 0.83% *vs.* 1.18 ± 0.83%, *p*=0.031); this difference was mainly observed at medium and high cholesterol levels at start of treatment (*p*=0.113, 0.051 and 0.041 at low, medium and high levels, respectively; Supplementary Fig. S4E). PCSK9, known to exert mild pro-inflammatory activity, was unchanged between treatment groups (p=0.768, Supplementary Fig. S4F). Although we observed some changes in TNF-α levels in low and medium cholesterol levels, there was no difference for high plasma cholesterol levels to explain our main results (Supplementary Fig. S5).

### 3.5 Colchicine reduces *in vitro* activation of circulating monocytes

**Fig. 5:**
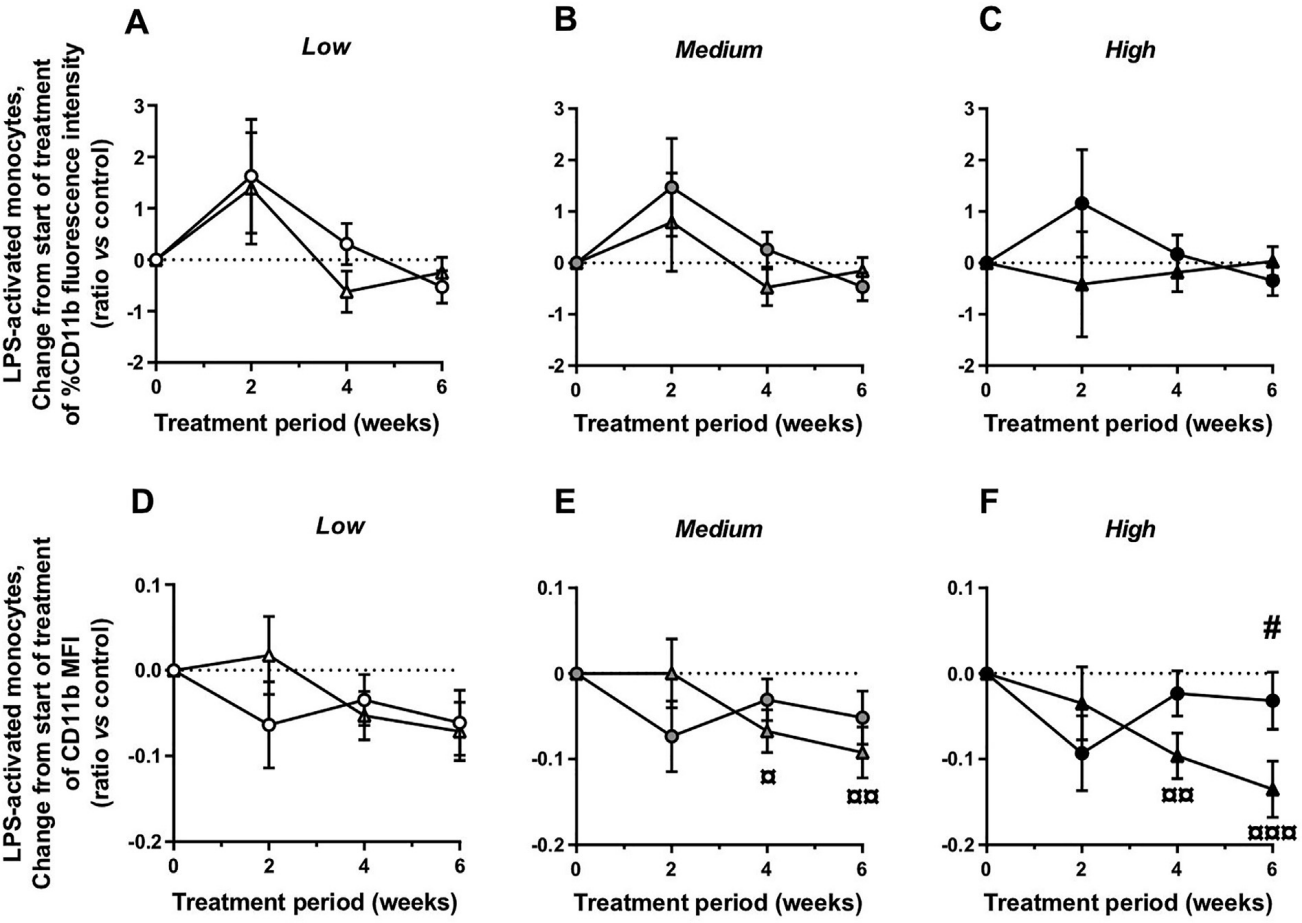
Colchicine reduces *in vitro* activation of circulating monocytes CD11b expression levels from rabbit whole blood samples were measured by flow cytometry analysis in monocyte sub-population after *in vitro* LPS stimulation (100 ng/mL). The expression of CD11b marker is presented as percent of monocyte population (A–C) and as median fluorescence intensity (MFI; D-F). Results are expressed as adjusted mean changes ± SE from start of treatment at each time points (2, 4 and 6 weeks after treatment) for placebo (circles, n=11) and colchicine-treated (triangles, n=11) groups and at specific values of plasma cholesterol at start of treatment (low, medium and high). ^**¤**^*p*<0.05, ^**¤¤**^*p*<0.01, ^**¤¤¤**^*p*<0.001, for change from start of treatment (2, 4 and 6 weeks after treatment *vs.* start of treatment); ^**#**^*p*<0.05, for colchicine *vs.* placebo.

The leucocyte activation assay was performed on rabbit blood samples obtained at weeks 10, 12, 14 and 16, by *in vitro* LPS stimulation (100 ng/mL). To determine the effect of LPS activation on monocytes and granulocytes, we analyzed the surface expression of activated CD11b, a member of the β2 integrin family, by flow cytometry. An LPS dose of 100 ng/mL induces a rapid increase in CD11b surface expression on monocytes in whole human blood [18]. In our rabbit blood samples at week 10 (before randomization), LPS stimulation resulted in a 2.8-fold increase of the percentage of CD11b^+^ monocytes and a 5.7-fold increase of CD11b^+^ granulocytes compared to control (unstimulated) samples, thus validating our stimulation protocol. During the treatment period, adjusted mean change from weeks 10–16 in percentage of CD11b^+^ cells in the monocyte and granulocyte populations was similar in both experimental groups (monocytes: *p*=0.534, 0.426 and 0.384 at low, medium and high cholesterol levels, respectively, Fig. 5A–C; granulocytes: *p*=0.547, 0.603 and 0.878 at low, medium and high cholesterol levels, respectively, data not shown). At high plasma cholesterol level at start of treatment, adjusted mean change from weeks 10–16 in median fluorescence intensity (MFI) for CD11b marker (indicating level expression of the surface marker per cell) in the monocyte population was significantly different between the colchicine group (˗0.135 ± 0.033, *p*<0.001) and the placebo group (−0.032 ± 0.033, *p*=0.353; *p*=0.041 between groups; Fig. 5D–F). A similar albeit non-significant trend was observed in the granulocyte population (colchicine *vs.* placebo: *p*=0.062; data not shown).

## 4 Discussion

Inflammation is an important component of atherosclerosis. Colchicine has been shown to reduce the risk of ischemic cardiovascular events in patients with coronary artery disease [[11], [12], [13]], but the underlying mechanisms are not completely understood. The present study shows that chronic oral colchicine treatment at a dose of 350 μg/kg/day decreases plaque vulnerability with reductions in plaque inflammation, medial fibrosis, outward vascular remodelling and circulating monocyte activation in an *in vivo* rabbit model of atherosclerosis.

Cholesterol-fed rabbits are widely used for atherosclerosis studies, mainly because of their human-like lipoprotein metabolism and their sensitivity to a cholesterol-enriched diet [19]. After a 10-week period of a 0.5% cholesterol-enriched diet, our rabbits developed hypercholesterolemia without elevation in plasma triglycerides, as previously reported by our group [20] and others [21]. At end of study, the thoracic aorta presented intimal atherosclerotic lesions characterized by macrophage (RAM-11^+^ cells) and T-cell (CD3^+^) infiltration, and by the presence of smooth muscle cells, lipid droplets and marked fibrosis. IVUS data revealed that these atherosclerotic lesions were associated with aortic vascular remodelling. Indeed, vessel volume and dimensions were increased, indicating outward (also called positive, expansive or compensatory) remodelling. In the current study, we showed that this remodelling was prevented by 6 weeks of treatment with colchicine and was associated with decreased plaque and media areas. In addition, previous studies reported that with progression of atherosclerosis, intimal smooth muscle cells exhibit reduced expression of SM2, and then SM1, whereas α-SMA was well preserved [22]. The observed phenotype change of vSMCs (higher SM2 marker expression with colchicine treatment *vs.* placebo) therefore also suggests favorable effects of colchicine on the atherosclerotic process.

Outward vascular remodelling, although limiting luminal narrowing, is considered a sign of plaque vulnerability and was reported being associated with poor clinical outcomes [[23], [24], [25], [26]]. This remodelling mainly occurs because of the growing plaque, and the extent of enlargement is positively correlated to plaque inflammation [27,28]. The precise mechanisms by which colchicine suppresses inflammation are still not fully understood. Colchicine inhibits cell microtubule assembly by preventing polymerization of tubulin monomers and modulates several leucocyte functions both *in vivo* (therapeutic doses) and *in vitro*.

In the current study, we showed that colchicine treatment decreased the presence of macrophages (RAM-11^+^ cells) in atherosclerotic plaques. Studies have shown that colchicine has direct anti-inflammatory effects by reducing activity of the NLRP3 inflammasome [29] and indirectly by a reduction of PCSK9 expression, known to exert a mild pro-inflammatory activity in atherosclerotic plaque [30]. We did not observe effects of colchicine on NLRP3, IL-6, IL-1β nor PCSK9 protein expression in plaques, suggesting that another anti-inflammatory mechanism was at play in our experimental model. However, we demonstrated that colchicine reduced activation of circulating monocytes in response to an *in vitro* LPS challenge, without reducing the proportion of activated cells. This mechanism by which colchicine reduces surface receptor expression was previously reported in neutrophils [31], T-cells [32] and platelets [33]. We hypothesize that this reduced sensitivity of monocyte to activation could result in decreases of monocyte adhesion and infiltration and might explain, at least in part, the reduction of macrophages in plaque observed in this study. Our results are in line with those of a recently published study of rabbits undergoing balloon denudation suggesting that colchicine could reduce vascular inflammatory activity measured by positron emission tomography [34].

Colchicine reduced the marked fibrosis exhibited by atherosclerotic lesions in the present study. Medial types I and III collagen expression was also decreased by the treatment with colchicine. This anti-fibrosis effect of colchicine could be associated with better elasticity of the aorta, as suggested by the increased elastin/type I collagen ratio. Colchicine has previously been shown to inhibit collagen synthesis and fibroblast proliferation [35,36]. In a rat model of cyclosporine nephrotoxicity, colchicine reduced fibrotic renal damage by reducing TGF-β overexpression, decreasing oxidative stress and suppressing renal cell apoptosis [37,38]. In rats with hypertensive chronic kidney disease, colchicine inhibited upregulation of fibronectin and type I collagen, and prevented SMAD3 phosphorylation, a canonical downstream mediator of TGF-β signaling [39]. In contrast, we did not observe any significant effect of colchicine on fibronectin, TGF˗β nor P-SMAD2/3 expression levels in arterial plaque or media.

A significant interaction between plasma cholesterol level at start of treatment and colchicine effects was observed in the current study. Indeed, the difference between treatment groups at end of study was not constant for all possible values of total cholesterol level at start of treatment. We believe that plasma cholesterol level is an important determinant of disease progression, thus allowing the effects of colchicine to be more readily detectable with more severe degrees of dyslipidemia.

### 4.1 Study limitations

Colchicine did not reduce the level of hs-CRP in the current study (Supplementary Fig. S6). In contrast, two weeks of treatment with colchicine 0.5 mg twice daily decreased this biomarker in an open-label pilot clinical study of 64 patients with stable coronary artery disease [40]. Although multiple benefits of colchicine were demonstrated with 6 weeks of treatment in the present study, including on vascular remodelling, longer duration of therapy and/or maintained exposure to 0.5% cholesterol diet during colchicine treatment might have resulted in more pronounced effects on the atherosclerotic process.

### 4.2 Conclusion

In rabbits with hypercholesterolemia-induced atherosclerosis, colchicine decreases plaque vulnerability with reductions in plaque inflammation, medial fibrosis, outward vascular remodelling and *ex vivo* monocyte activation. These results provide potential mechanisms for the clinical benefits of colchicine in the prevention of atherothrombotic events in patients with coronary artery disease.

## Financial support

Dr. Tardif holds the Canada Research Chair in translational and personalized medicine and the Université de Montréal Pfizer-endowed research chair in atherosclerosis. The work was funded by the Health Collaboration Acceleration Fund from the Government of Quebec.

## Credit authorship contribution statement

François Roubille: wrote the manuscript, conducted the experiments, and designed the experiments. Nolwenn Merlet: wrote the manuscript, conducted the experiments. David Busseuil: conducted the experiments. Marine Ferron: conducted the experiments. Yanfen Shi: conducted the experiments. Teodora Mihalache-Avram: conducted the experiments. Mélanie Mecteau: conducted the experiments. Geneviève Brand: conducted the experiments. Daniel Rivas: conducted the experiments. Mariève Cossette: performed statistical analyses. Marie-Claude Guertin: performed statistical analyses. Éric Rhéaume: designed the experiments. Jean-Claude Tardif: designed the experiments. All authors reviewed the manuscript.

## Declaration of competing interest

The authors declare the following financial interests/personal relationships which may be considered as potential competing interests: Dr. Tardif reports receiving grant support from Amarin, Esperion, Ionis PharmaceuticalsIonis Pharmaceuticals and RegenXBio, receiving grant support and honoraria from AstraZeneca, Pfizer and Sanofi, receiving grant support and honoraria from and having minor equity interest in DalCor PharmaceuticalsDalCor Pharmaceuticals, holding a pending patent (US20170233812A1) on genetic markers for predicting responsiveness to therapy with a high-density lipoprotein (HDL)–raising or HDL mimicking agent, and holding pending patents (62/935,751 and 62/935,865) on methods for using low-dose colchicine after myocardial infarction, licensed to Montreal Heart Institute (Dr. Tardif has waived his rights in colchicine patents and does not stand to gain financially).
