# Supplementary material for: Colchicine reduces atherosclerotic plaque vulnerability in rabbits
Source: Atheroscler Plus. 2021 Sep 2;45:1–9. doi: 10.1016/j.athplu.2021.08.008 (PMC9833268; doi:10.1016/j.athplu.2021.08.008)

**Supplementary methods**

**Colchicine reduces atherosclerotic plaque vulnerability in rabbits**

François Roubille, Nolwenn Merlet, David Busseuil, Marine Ferron, Yanfen Shi, Teodora Mihalache-Avram, Mélanie Mecteau, Geneviève Brand, Daniel Rivas, Mariève Cossette, Marie- Claude Guertin, Eric Rhéaume, Jean-Claude Tardif

Table of contents

1. [Animal studies 2](#_bookmark0)
2. [Intravascular ultrasound (IVUS) imaging 2](#_bookmark1)
3. [Immunohistochemistry 3](#_bookmark2)
4. [Plasma analyses 4](#_bookmark3)
5. [In vitro LPS challenge of blood cells 5](#_bookmark4)
6. [Statistical analyses 5](#_bookmark5)
7. [Supplementary Tables 7](#_bookmark6)
8. [Supplementary Figures 9](#_bookmark7)

# Animal studies

Colchicine was purchased from *Sigma-Aldrich*, aliquoted following the manufacturer’s recommendations and kept at -20°C. The individual doses for each rabbit were prepared each day by experienced operators, mixed in yogurt and administered at the same hour every day; the first dose of colchicine was given at week 10, on the day after the IVUS procedure. The operators were all unaware of the treatment allocation except for the operator responsible for the feeding.

One rabbit died before the start of treatment during the IVUS procedure and two rabbits (one in the placebo group, and one in the colchicine group) died during the IVUS procedure at end of treatment.

# Intravascular ultrasound (IVUS) imaging

IVUS images were obtained using an Atlantis® SR Pro Coronary Imaging catheter with a central frequency of the rotating transducer of 40 MHz (*Boston Scientific Corporation*). Procedures were performed at weeks 10 and 16 under ketamine induction (35 mg/kg, i.m.) followed by isoflurane (*Abbot*, Montreal, Canada) administration (induction 2.5-3%, 0.7 L/min oxygen, adapted to heart rate, breath rate). To maintain analgesia, buprenorphine (10 µg/kg, s.c., *Schering-Plough*, Hertfordshire, UK) was administered before surgery and systematically repeated once daily for 3 days after surgery. Post-surgical analgesia was maintained using buprenorphine titratedevery 6-12 hours during 48 hours, if necessary. Thermoregulation was achieved during the procedure.

Arterial access was obtained *via* the right (first procedure, week 10) or the left (second terminal procedure, week 16) common carotid artery. The carotid artery was exposed and a radial introducer (*Cordis*) was inserted. The artery was occluded distally to the introducer. The IVUS

catheter was introduced through the radial introducer over a 0.014-inch coronary guidewire (*Abbot*) and placed at the iliac bifurcation. An automated pullback of the IVUS catheter was performed in the descending thoracic aorta, using a pullback device (Boston Scientific Corporation) at a speed of 0.5 mm/s and a frame rate of 30 images/second. Experiments were all performed by an experienced team, and all interventions were carried out by a single operator.

IVUS analyses were performed by an experienced observer blinded to group assignment. For each rabbit, alignment of pullbacks at weeks 10 and 16 was obtained using fixed anatomical landmarks to analyze the same segment at the 2 time-points. IVUS cross-sections were selected at every 60th image along the vessel’s longitudinal axis. Vessel area, defined as that circumscribed by the external elastic membrane, and lumen area were traced at each selected cross-section using the EchoPlaque software version 4.0.19 (*INDEC Medical Systems*, Santa Clara, CA, USA). Vessel and lumen volumes were computed by the summation of the respective areas over all traced cross- sections of the segment, and results were then indexed to a 30-mm segment. Plaque volume was obtained by subtracting lumen volume from vessel volume.

# Immunohistochemistry

Sections were fixed in acetone (˗20°C, 10 minutes) and washed in phosphate buffered saline (PBS), except for RAM-11, IL˗1β and NLRP3 where sections were fixed in 4% paraformaldehyde (4°C, 10 minutes), washed in phosphate buffered saline (PBS) and then incubated with antigen retrieval solution (Retriever, *Vector Laboratories*, Burlington, ON, Canada) for 30 minutes. All sections were treated for 5 minutes with 3% H2O2 to block endogenous peroxidase activity, and then washed in PBS. The sections were then treated for 20 minutes at room temperature with standard blocking serum (10% (or 20% for VCAM-1 and ICAM-1) normal horse or goat serum in PBS supplemented with 0.1% Triton X-100), before being incubated with respective primary

antibodies, overnight, at 4°C (see Supplemental Table S1 for details of primary antibodies). Sections were then washed in PBS and incubated with appropriate biotinylated secondary antibody (goat anti-mouse IgG [BA9200, *Vector Laboratories*], goat anti-rat IgG [BA9400, *Vector Laboratories*], horse anti-goat IgG [BA9500, *Vector Laboratories*] or goat anti-guinea-pig IgG [BA7000, *Vector Laboratories*]), at a dilution of 1/200 for 1 hour (75 minutes for CD3 staining) at room temperature. Secondary antibody incubations were followed by Vectastain Elite ABC kit (*Vector laboratorie*s) for 30 minutes at room temperature. Finally, section staining was revealed with a Romulin AEC chromogen kit (*Biocare Medical*, Concord, CA, USA), counterstained with hematoxylin (Sigma Aldrich, Oakville, ON, Canada), dehydrated by successive ethanol (70%, 95% and 100%) and toluene baths and then mounted on Permount (*Fisher Scientific*, Toronto, ON, Canada). Percentage of antibody positive staining area in selected regions of interest was assessed on section pictures.

All pictures were taken with a computer-based digitizing image system using a light microscope (*Olympus* BX41, Richmond Hill, ON, Canada) connected to a digital video camera Q- color3 (*Olympus*, Richmond Hill, ON, Canada). Image Pro Plus version 7.0 and higher (*Mediacybernetics*, Bethesda, MD, USA) were used for picture acquisition and analysis.

# Plasma analyses

Levels of high-sensitivity C-reactive protein were measured during the treatment period by enzyme-linked immunosorbent assay (ELISA) according to manufacturer’s instructions (GWB- 9BF960, *Genway Biotech*, San Diego, CA, USA). Total cholesterol, triglycerides, alanine aminotransferase (ALT), aspartate aminotransferase (AST), gamma-glutamyl transferase (GGT)

and creatinine levels were measured with an automated filter photometer system (Dimension RxL Max; *Dade Behring*, Deerfield, IL, USA).

1. ***In vitro* LPS challenge of blood cells**

Whole blood samples collected on citrate-coated tubes were incubated with LPS (100 ng/mL) for 15 minutes at 37°C, and then stained with rat anti-mouse CD11b antibody (clone M1/70.15, conjugated to Alexa Fluor 488, 1/10 dilution, MCA74A488, *AbD Serotec*) for 30 minutes at 4°C. FACS lysing solution (BD Pharm Lyse, 555899, *BD Biosciences*) was added for 15 minutes at room temperature and samples were stored at 4°C until analysis by flow cytometry (LSRII BD Biosciences). Data were analyzed on Diva software version 5.0.3 (*BD*).

# Statistical analyses

For the 6-week treatment period (weeks 10 to 16), outcomes expressed as mean changes from week 10 at different time points (weeks 12, 1S4 and 16) were analyzed using a repeated measures ANCOVA model including terms for group, time and group-by-time interaction as well as covariates for response variable at week 10, response variable at week 10-by-time-interaction, total cholesterol at week 10 and total cholesterol at week 10-by-time-interaction; adjustment for plasma total cholesterol at week 10 was chosen because this variable presented intra-group heterogeneity (Supplemental Fig. S1) that could affect atherosclerosis plaque development. An unstructured covariance structure was used to model the within-subject errors and the Kenward- Roger approximation was used to estimate denominator degrees of freedom. The primary comparison was the contrast between groups at the last follow-up time point (week 16).

Outcomes expressed as mean changes from weeks 10 to 16 only were analyzed using an ANCOVA model including a term for group as well as covariates for response variable at week 10 and total cholesterol at week 10. Outcomes measured after the sacrifice were compared between groups using an ANCOVA model adjusted for total cholesterol at week 10. In an exploratory

fashion, total cholesterol at week 10 by treatment interaction was added to the ANCOVA models, even when non-significant, and comparisons between groups were tested at specific values of cholesterol at week 10. The specific cholesterol values at start of treatment were chosen as the quartiles (Q1 [11.20 mM; low level], median [13.43 mM; medium level] and Q3 [17.95 mM; high level]). However, these values are not used to classify rabbits into groups. An adjusted mean had been computed for a specific value of the covariate (cholesterol at start of treatment) and this adjusted mean (change in the outcome from start of treatment) is an estimate of the response of a typical rabbit that would have this value of cholesterol at start of treatment.

1. **Supplementary Tables Supplementary Table S1**

**Antibodies and conditions used for immunohistochemistry.**

|  | **Primary Antibody** | **Clone** | **Reference** | **Dilution** |
| --- | --- | --- | --- | --- |
| **Macrophages** | Mouse anti-rabbit macrophage | RAM-11 | M0633, *DakoCytomation*, Burlington, ON, Canada | 1/100, in  1% NGS |
| **T-cells** | Rat monoclonal anti- | CD3-12 | MCA1477, *Abd Serotec - Bio-Rad* | 1/100, in |
|  | human CD3 |  | *Laboratories*, Hercules, CA, USA | 1% NGS |
| **Neutrophils** | Mouse anti-rabbit T- cells and neutrophils | RPN3/57 | MCA805GA, *Abd Serotec - Bio- Rad Laboratories*, Hercules, CA, | 1/100, in  1% NGS |
|  |  |  | USA |  |
| **Smooth muscle cells** | Mouse anti-human smooth muscle actin | 1A4 | M0851, *DakoCytomation*, Burlington, ON, Canada | 1/500, in  1% NGS |
| **SM1** | Mouse anti-smooth muscle myosin heavy chain I | 1C10 | ab681, *Abcam*, Cambridge, MA, USA | 1/3000, in  1% NGS |
| **SM2** | Mouse anti-smooth muscle myosin heavy chain II | 1G12 | ab683, *Abcam*, Cambridge, MA, USA | 1/400, in  1% NGS |
| **IL-6** | Guinea-pig anti-rabbit interleukin 6 | *Polyclonal* | PAA079Rb51, *Cloud-Clone Corp.* -  *Cedarlane*, Burlington, ON, Canada | 1/20, in  1% NGS |
| **IL-1β** | Cavia anti-rabbit | *Polyclonal* | MBS2001442, *MyBiosource*, San | 1/20, in |
|  | interleukin 1 beta |  | Diego, CA, USA | 1% NGS |
| **NLRP3** | Goat anti-human | *Polyclonal* | MBS241659, *MyBiosource*, San | 1/100, in |
|  | NLRP3 |  | Diego, CA, USA | 1% NHS |
| **VCAM-1** | Mouse anti-CD106 | RB1/9 | kindly provided by Pr Miron Cybulsky, University of Toronto, | 1/10, in  1% NGS |
|  |  |  | Canada |  |
| **ICAM-1** | Mouse anti-CD54 | RB2/3 | kindly provided by Pr Miron Cybulsky, University of Toronto, | 1/100, in  1% NGS |
|  |  |  | Canada |  |
| **Type I collagen** | Goat anti-human type I collagen | *Polyclonal* | AB758, *EMD Millipore*, Chicago, IL, USA | 1/800, in  1% NHS |
| **Type III collagen** | Mouse anti-human type III collagen | FH-7A | ab6310, *Abcam*, Cambridge, MA, USA | 1/4000, in  1% NGS |
| **MMP9** | Rat anti-mouse matrix metalloproteinase 9 | 116134 | BAM9091, *R&D Systems*, Minneapolis, MN, USA | 1/100, in  1% NGS |
| **TGF-β1** | Mouse anti-human | TB21 | ab27969, *Abcam*, Cambridge, MA, | 1/200, in |

transforming growth factor beta 1

USA

1% NGS

| **Fibronectin** | Mouse | anti-human | IST-3 | F0791, *Sigma-Aldrich*, Oakville, | 1/100, in |
| --- | --- | --- | --- | --- | --- |
|  | fibronectin |  |  | ON, Canada | 1% NGS |

| **P-SMAD2/3** | Goat anti-human phosphorylated Smad2/3 (Ser423/425) | *Polyclonal* | Sc-11769, *Santa Cruz*, Mississauga, ON, Canada | 1/100, in  1% NHS |
| --- | --- | --- | --- | --- |
| **PCSK9** | Goat anti-mouse and human Proprotein Convertase 9 | *Polyclonal* | AF3985, *R&D Systems*, Minneapolis, MN, USA | 1/100, in  1% NHS |

NGS: normal goat serum, NHS: normal horse serum.

**Supplementary Table S2**

**Circulating values for total cholesterol and triglyceride levels.**

|  | | **Placebo (n=11)** | **Colchicine (n=11)** | ***P-Value*** |
| --- | --- | --- | --- | --- |
|  | **Before treatment** | 15.1 ± 5.6 | 14.8 ± 6.3 | *0.917* |
| **Total cholesterol** | **Treatment Week 2** | 14.9 ± 1.7 | 12.9 ± 1.7 | *0.409* |
| **(mM)** | **Treatment Week 4** | 13.0 ± 1.4 | 12.5 ± 1.4 | *0.792* |
|  | **Treatment Week 6** | 13.3 ± 1.4 | 11.6 ± 1.4 | *0.399* |
|  | **Before treatment** | 1.0 ± 0.5 | 1.0 ± 0.5 | *0.884* |
| **Triglycerids (mM)** | **Treatment Week 2**  **Treatment Week 4** | 1.24 ± 0.29  0.92 ± 0.21 | 1.41 ± 0.29  1.33 ± 0.21 | *0.687*  *0.184* |
|  | **Treatment Week 6** | 0.97 ± 0.17 | 1.02 ± 0.17 | *0.845* |

# Supplementary Figures

# Supplementary Fig. S1. Total plasma cholesterol levels at start of treatment.

Results are expressed as individual plots and median ± interquartile range for placebo (n=11) and colchicine (n=11) groups.


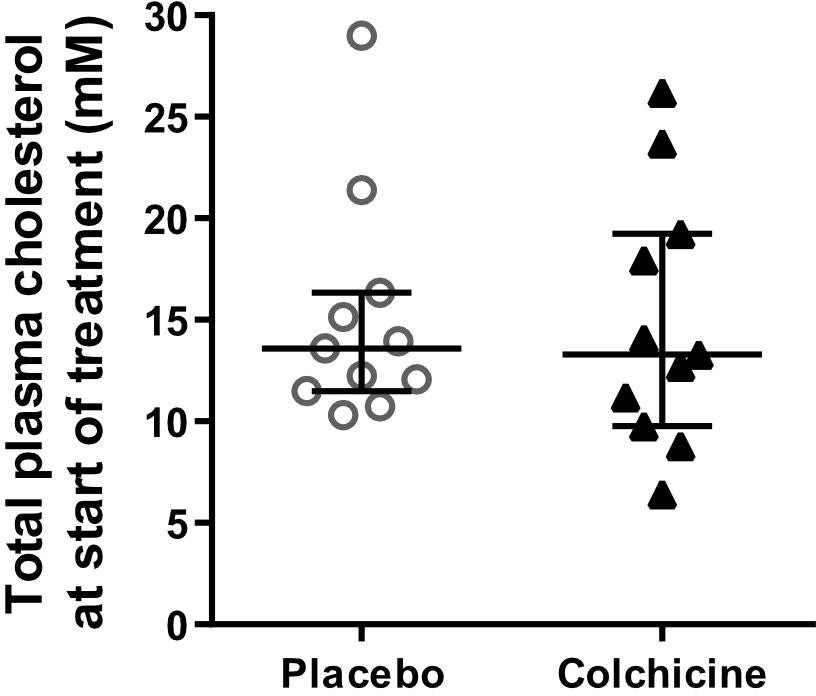


**Supplementary Fig. S2. Rabbit body weight curves of placebo and colchicine-treated rabbits.** Rabbits were fed a 0.5% cholesterol-enriched diet from weeks 0 to 10. During treatment period, from weeks 10 to 16, diet was reduced to a 0.2% cholesterol-enriched diet. At start and end of treatment, rabbits were imaged by intravascular ultrasound (IVUS).


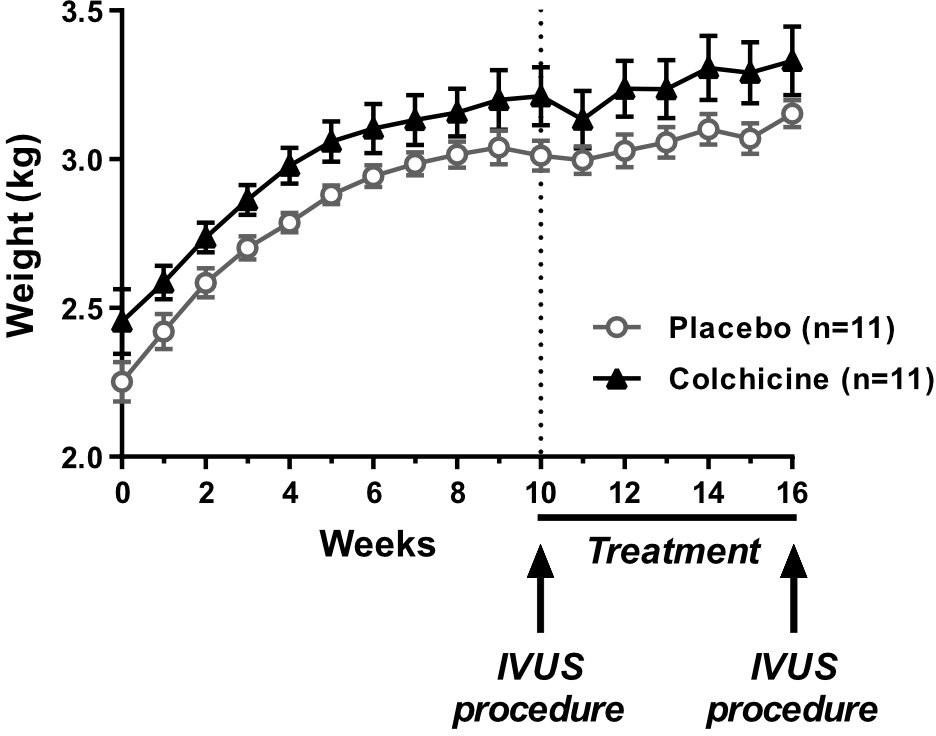


at specific values of plasma cholesterol at start of treatment (low, medium and high). Pictures are representative staining at high cholesterol value at start of treatment. Scale bars represent 100 µm. **#** *p*<0.05, for colchicine *vs.* placebo.


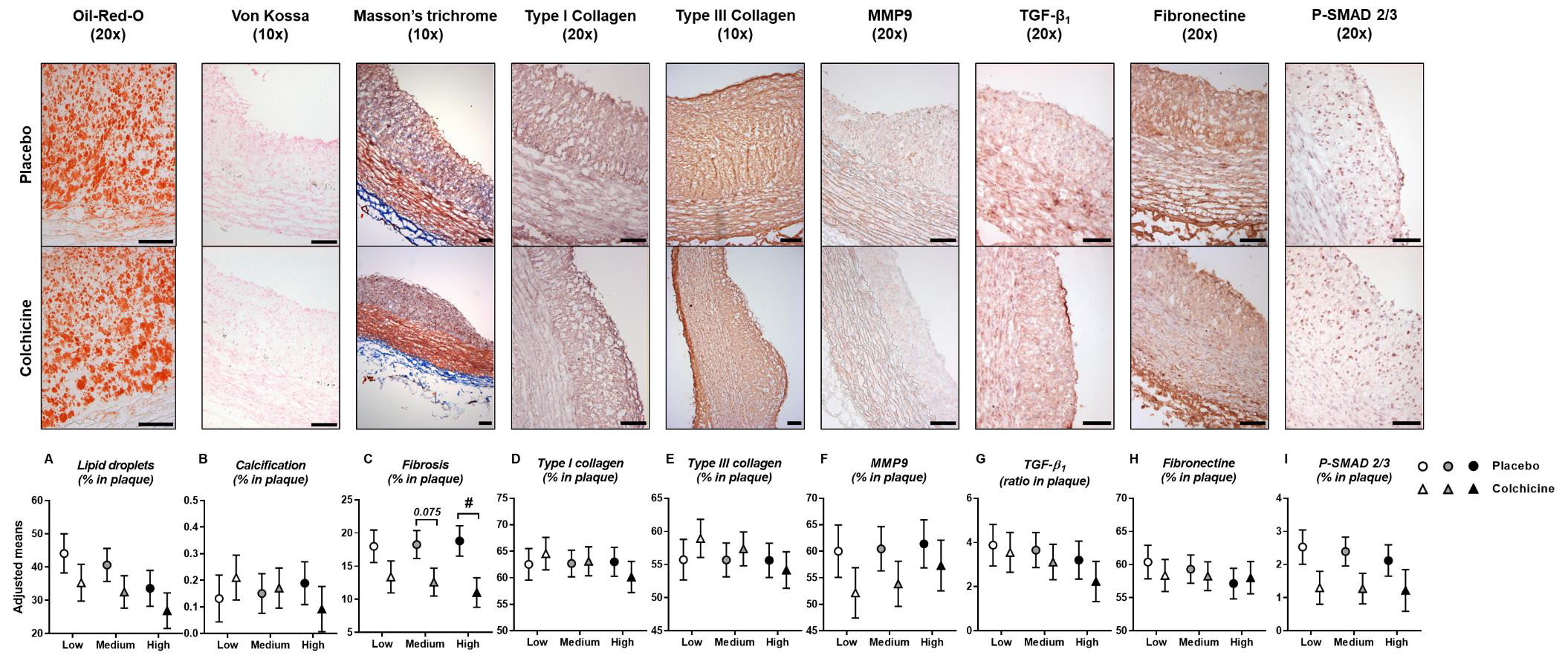


**Supplementary Fig. S4. Inflammation markers in atherosclerotic plaques.**

Results are expressed as adjusted means ± SE of each staining for placebo (circles, n=11) and colchicine-treated (triangles, n=11) groups at specific values of plasma cholesterol at start of treatment (low, medium and high). Pictures are representative staining obtained at high plasma cholesterol value at start of treatment. Scale bars represent 100 µm. **#** *p*<0.05, for colchicine *vs.* placebo.


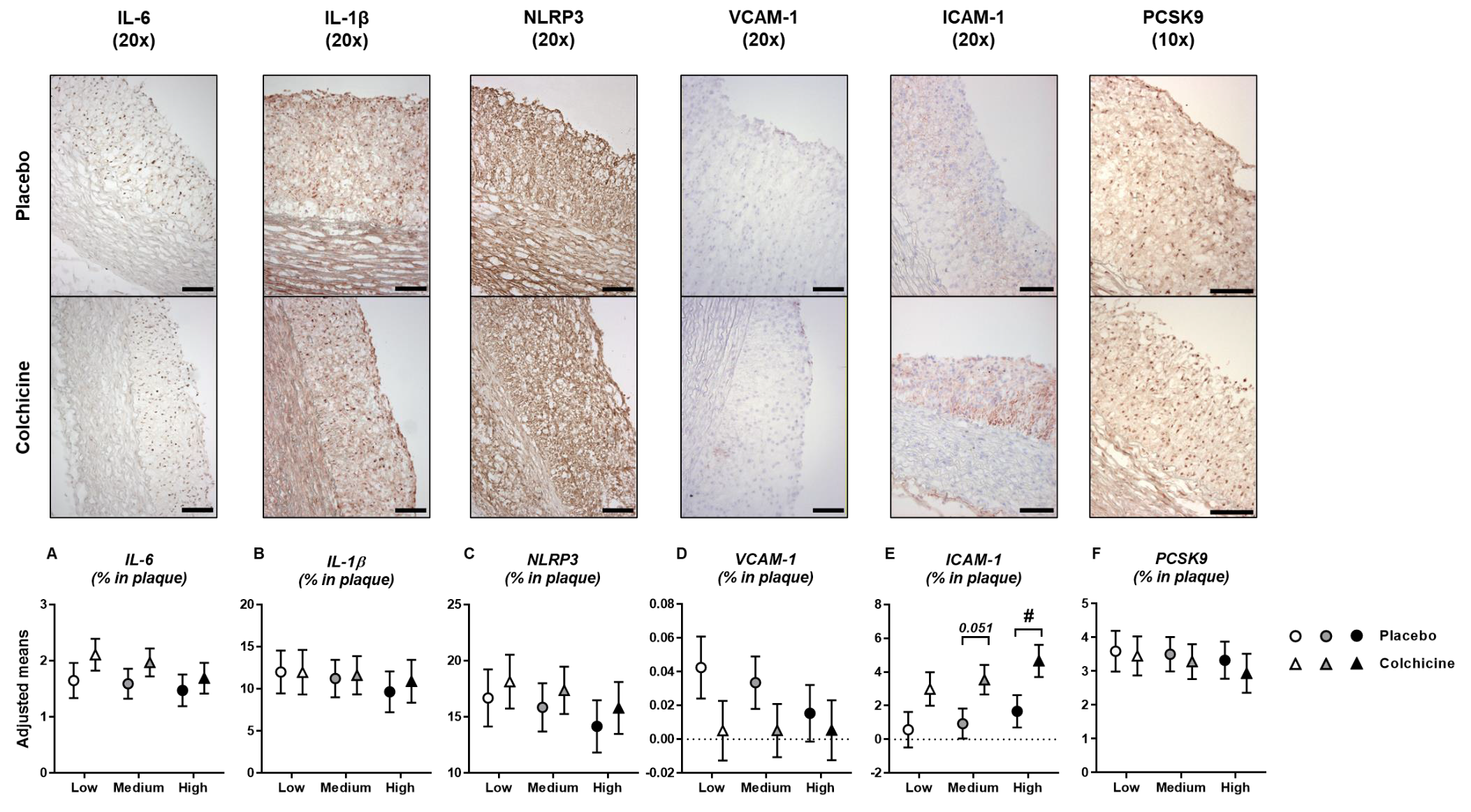


**Supplementary Fig. S5. Circulating TNF-α levels.**

Results are expressed as adjusted mean changes ± SE from start of treatment at each time point (2, 4 and 6 weeks after treatment) for placebo (circles) and colchicine-treated (triangles) groups and at specific values of plasma cholesterol at start of treatment (low, medium and high). ¤ p<0.05, for change from start of treatment (2, 4 and 6 weeks after treatment *vs*. start of treatment), * p<0.05, for colchicine *vs.* placebo.


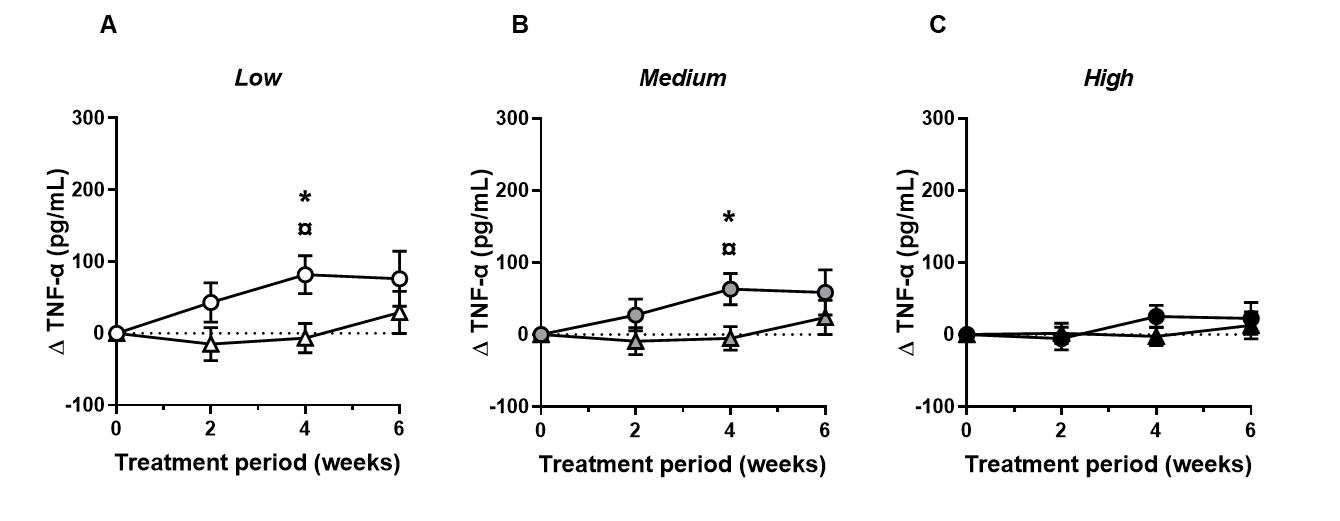


**Supplementary Fig. S6. Colchicine does not reduce circulating CRP levels.**

Results are expressed as adjusted mean changes ± SE from start of treatment at each time points (2, 4 and 6 weeks after treatment) for placebo (circles) and colchicine-treated (triangles) groups and at specific values of plasma cholesterol at start of treatment (low, medium and high). **¤** *p*<0.05, for change from start of treatment (2, 4 and 6 weeks after treatment *vs.* start of treatment).


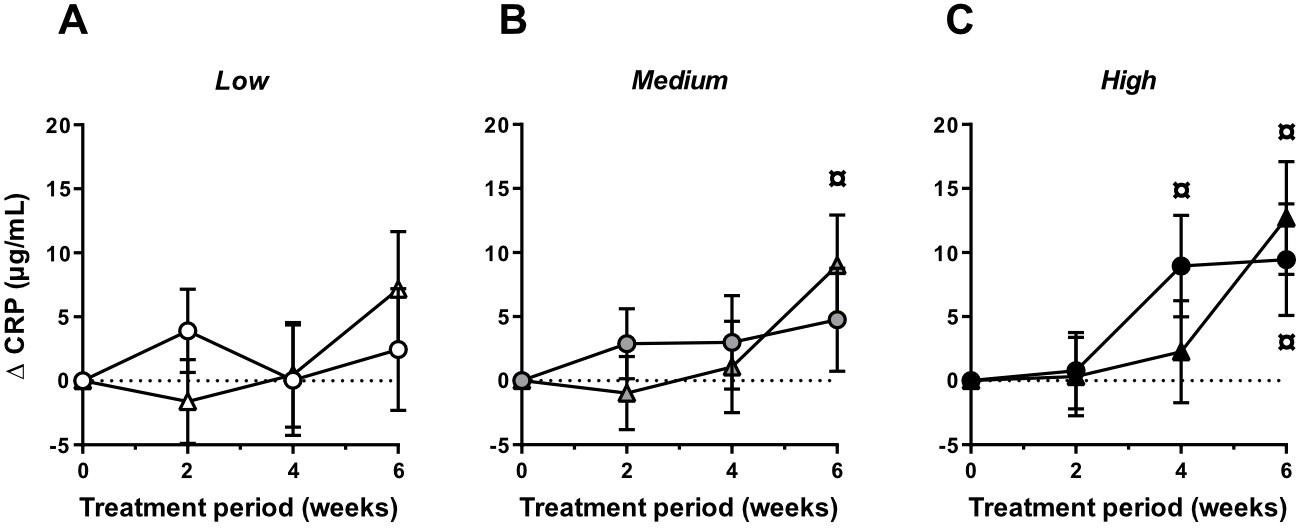

Supplement: Multimedia component 1 [file mmc1.docx]
